# Assessing the impact of immobilisation on the bioavailability of PFAS to plants in contaminated Australian soils

**DOI:** 10.1007/s11356-024-32496-7

**Published:** 2024-02-19

**Authors:** Sali Khair Biek, Leadin S. Khudur, Laura Rigby, Navneet Singh, Matthew Askeland, Andrew S. Ball

**Affiliations:** 1 ARC Training Centre for the Transformation of Australia’s Biosolids Resource, RMIT University, Bundoora, VIC 3083 Australia; 2 School of Science, STEM Collage, RMIT University, Bundoora, VIC 3083 Australia; 3 ADE Consulting Group Pty Ltd, Williamstown North, VIC 3016 Australia

**Keywords:** PFAS, Leachability, Bioavailability, Ecotoxicity, Immobilisation, Immobilising agent

## Abstract

Per- and polyfluoroalkyl substances (PFAS) have become a key concern to both environmental and human health due to their extreme persistence in the environment and their ability to bioaccumulate in plants, animals, and humans. In this mesocosm study, Australian PFAS-contaminated soil with a mean total concentration of 8.05 mg/kg and a mean combined PFHxS + PFOS concentration of 7.89 mg/kg was treated with an immobilisation sorbent (RemBind®) at different application rates (0.5, 1, 1.5, 2, 3, 4, and 5% w/w). To assess the efficacy of this immobilisation treatment, PFAS leachability, PFAS plant uptake, and ecotoxicity tests were conducted. Leachability testing was performed according to the Australian Standard Leaching Procedure (ASLP) at pH 5 and 7. A grass species (*Dactylis glomerata*) was used to measure plant uptake of PFAS from untreated and treated contaminated soil. In addition, the Microtox test was used to assess the associated ecotoxicity. The immobilisation treatment resulted in a significant reduction of 88.5–99.8% in the total PFAS leachability and 88.7–99.8% in the combined PFOS and PFHxS leachability at pH 5. Similarly, significant reductions (5–12-fold) were observed in the plant uptake of total PFAS and combined PFOS and PFHxS in all treated soil samples. In addition, although the Microtox test showed relatively low ecotoxicity in all the experimental samples, including the untreated soil, a significant decrease in the ecotoxicity of treated soil samples was observed. The results from this study highlight that this treatment approach has the potential to reduce both PFAS leachability and plant bioavailability with a relatively low associated ecotoxicity. This is likely to reduce the risk of the transfer of PFAS into higher trophic levels. This immobilisation treatment may, therefore, reduce the risk associated with PFAS-contaminated soils and may be an important remediation tool for managing certain PFAS-contaminated soils.

**Graphical Abstract:**

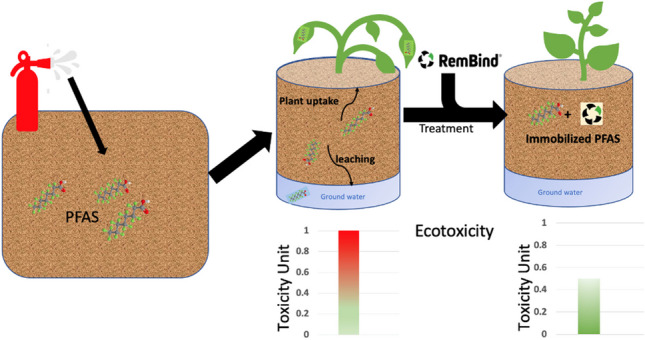

**Supplementary Information:**

The online version contains supplementary material available at 10.1007/s11356-024-32496-7.

## Introduction

Per- and polyfluoroalkyl substances (PFAS) are a large and complex group of more than 4000 synthetic (man-made) organofluoride chemicals that belong to a family of highly fluorinated aliphatic compounds with chemical structure generally formulated as “C_n_F_2n+1_ − R” (Buck et al. 2011; Mueller and Yingling 2017). Perfluoroalkyl substances are stable at high temperatures, non-flammable, non-degradable, and are not subject to photolytic degradation; fluorine atoms have replaced all the hydrogen atoms bonded to carbon in the linear carbon chain. In contrast, in polyfluoroalkyl substances, the backbone still contains hydrogenated carbon atoms, although the molecule remains highly fluorinated (Lau et al. 2004).

Perfluorooctane sulfonic acid (PFOS) and perfluorooctanoic acid (PFOA) represent the most commonly used perfluoroalkyl substances (Becker et al. 2010). They are classified in the Stockholm Convention as persistent organic pollutants (POPs) for their high persistence, toxicity, ability to bioaccumulate, and abundant environmental distribution. They represent a high proportion of PFAS environmental contamination (Fiedler and Sadia 2021; UNEP 2009; OECD 2013).

Due to their desirable physicochemical properties, PFAS have been used to provide key properties to a product, including stain and soil resistance (Banks et al. 2013; Rao and Baker 1994), grease-proofing (Rice 2015), surface tension reduction (as effective surfactants or surface protectors), and impermeability to water and non-stick surface (Knepper and Lange 2011). In addition, due to their ability to create stable foams (Kempisty et al. 2016), PFAS were, until recently, widely used as the main components of aqueous film-forming foam (AFFF) at military bases, oil refineries, and airports for fire suppression (Wang et al. 2017; Moody and Field 2000). The uses of PFAS based on their useful chemical properties is, paradoxically, the reason why PFAS pose a problem in the environment, namely, their long-term persistence in the environment and consequently in living organisms. They are often referred to as “forever chemicals” (Allen 2018).

Per- and polyfluoroalkyl substances have infiltrated everyday life and activities; although they do not occur naturally, their widespread use and release into the wastewater system have resulted in their appearance in the environment through those activities (Moodie et al. 2021). Per- and polyfluoroalkyl substances can affect the soil biota and can be taken up by plants (Sima and Jaffé, 2021). The leachate from the PFAS-contaminated soil can also enter surface and ground waters (Barzen-Hanson et al. 2017). They are released into the environment (both aquatic and terrestrial) during and after the production and usage of PFAS-containing products, exposing the biota in the environment to PFAS (Ahrens and Bundschuh 2014). In both cases, PFAS can magnify along the food chain to a level that poses a risk to humans.

In recent years, the human health implications relating to the global distribution of PFAS and their accumulation in the environment and human tissues have been recognised, as many harmful health effects have been reported linked to the exposure of several types of PFAS (Jian et al. 2018; Stubleski et al. 2016). Per- and polyfluoroalkyl substances have not only various adverse effects on human health but also their abundant presence in the environment, such as soil and biosolids, negatively affect the soil biota, limiting ecosystem function and services through the induction of oxidative stress that affects their behaviour and survival (Yuan et al. 2017). In addition, due to their strong affinity to proteins (Cheng and Ng 2018; Loi et al. 2011), PFAS can bioaccumulate and biomagnify throughout the food chain following uptake by plants and animals. Therefore, it is necessary to understand the behaviour of PFAS in the soil–plant system in contaminated soil as well as remediated soil to assess the effectiveness of any treatment approach.

To assess the associated ecotoxicity of contaminated soil and the efficacy of any treatment, several ecotoxicological tests and procedures have been used to quantify the overall toxic effect on selected test organisms such as fish, worms, mice, algae, and bacteria. The disadvantages of tests that use animal bioassay are the organisms’ standardisation, requirements for special equipment and skilled operators, lack of reproducibility, and long-duration assays. Bacterial bioassays, including the Microtox toxicity test, which has been approved as a standard test to assess the ecotoxicity of a variety of contaminants are relatively quick and simple (Dutka and Kwan 1981; De Zwart and Slooff 1983; Standard 1997; Domínguez et al. 2023). The Microtox toxicity test uses *Aliivibrio fischeri*, a marine bioluminescent bacterium formerly known as *Photobacterium phosphoreum* (Kamlet et al. 1986).

The widespread distribution and persistence of PFAS in the environment and throughout ecosystems indicate an urgent need for the development of an efficient method to remediate PFAS. The existence of PFAS as complex mixtures in environmental matrices and their extremely persistent nature make the remediation of PFAS in both aqueous and solid/sediment extremely challenging.

Recently, immobilisation approaches have been used to treat PFAS-contaminated soil using soil sorbents or fixation agents. Adding sorbents redistributes PFAS contaminants, reducing their concentration in the liquid phase, thereby decreasing their mobility, leachability, and bioavailability (Hale et al. 2017; Sleep and Juhasz 2021; Juhasz et al. 2022a). The application of immobilising agents such as RemBind® which is a commercial adsorbent containing activated carbon, clays, and aluminium oxyhydroxides has been applied to a wide range of soils contaminated with PFAS (Bräunig et al. 2021a). RemBind® work through adsorption mainly by forming electrostatic interactions between anionic PFAS and aluminium hydroxide and also through the hydrophobic interactions and van der Waals forces with activated carbon and organic matter components (Juhasz et al. 2022a).

Immobilisation (stabilisation) of PFAS in contaminated soil is considered an efficient, cost-effective, and robust treatment that can be applied both in situ and ex situ to remediate contaminated soils in comparison to other remediation approaches (Mahinroosta and Senevirathna 2020). However, although immobilisation reduces the PFAS contaminants from leaching, it does not destroy the contaminants. Therefore, the long-term efficiency and stability of the immobilisation process are critical for future study; in addition, assessment of the impact of immobilisation on the risk that the contaminated soils pose is important.

Soils serve as a major source of PFAS which can leach into groundwater and surface water and cause harm to human health and the environment. It is, therefore, important to understand the dynamics of PFAS contaminants in the environment and their fate and to assess the impact of any remediation approach on the availability of PFAS to receptors such as plants. Despite the abundance of data on the effectiveness of soil amendments in decreasing the leachability of PFAS, few studies have been conducted on their impact in reducing PFAS bioavailability for ecological and/or human receptors (Bräunig et al. 2021b). Juhasz et al., in their study, highlighted the effect of soil amendments on reducing both PFAS leachability by 98% and relative bioavailability using in vivo mouse bioassay by 60.5–87.5% (Juhasz et al. 2022a).

Also, very limited studies assess the potential of soil amendments to reduce PFAS exposure in soil invertebrates and higher organisms; however, studying the effect of immobilisation using the Microtox test is yet to be undertaken. This is important as soil amendment efficacy may vary in higher organisms due to their complexity and the effect of multiple factors; the Microtox test offers a standardised test that might be beneficial for long-term and stability studies.

The aim of this study was to assess the efficiency of the PFAS immobilisation process in contaminated Australian soil by assessing the reduction in PFAS leachability, plant uptake, and the associated ecotoxicity using a Microtox test of the soil following immobilisation using a common sorbent at different application rate.

## Materials and methods

### Chemicals and reagents

All chemicals and reagents used in this study were sourced from Rowe Scientific Pty Ltd, Melbourne, Victoria, unless otherwise stated. Glassware pots were cleaned before use to reduce background PFAS contamination following the “Decontamination Procedure for Equipment used for Sample Collection for Per- and Poly-Fluoroalkyl Substances (PFAS)” as outlined by the EPA (USEPA 2019) simply by rinsing with warm water; scrubbing with a low-phosphate detergent; rinsing three times with tap, and then deionized water, followed by a triple methanol rinse; and air drying.

### Soil samples

Around 13 kg of soil was collected from an aqueous film-forming foam (AFFF)-impacted site in Australia and provided by ADE Consulting Group Pty Ltd. The soil was known to be impacted by PFAS contamination and considered site-representative. Soil characterisation identified the soil as sandy clay soil. Prior to any further analysis, the soil was homogenised using an end-over-end shaker. Sub-samples of the homogenised soil were taken for further experiments and analyses, including total PFAS concentration, treatment application, leachability, plant bioavailability, and ecotoxicity. All soil treatments and analyses were performed in triplicate.

### The application of the immobilising agent

RemBind® (RB), the immobilising sorbent used in this study, was sourced from an environmental remediation products company (RemBind® Pty Ltd., South Australia) and used according to the supplier’s recommendations.

Eight soil samples with a total weight of 1 kg each were prepared, and for each treatment, a suitable amount of soil was added to a 1-L PFAS-free container. RemBind® was then added to the soil samples except for one (untreated) soil sample, and a series of bench trials were conducted by treating the contaminated soil with applications of 0.5, 1.0, 1.5, 2.0, 3.0, 4.0, and 5.0% (w/w) RB. Samples were well mixed by tumbling on an end-over-end shaker for 1 h and then transferred to PFAS-free high-density polyethylene (HDPE) jars for further lab analysis and divided into three replicates. All equipment used was rinsed with standard reagent grade methanol, followed by PFAS-free MilliQ water to prevent cross-contamination.

### Plant

To assess the plant uptake of PFAS from the contaminated soil, *Dactylis glomerata*, commonly known as cock’s-foot, orchard grass, or cat grass, was selected. This plant species is easy to plant, has shown good adaptation and growth for a small-scale experiment, and has been used in prior studies on plant uptake grown on contaminated soil (Tatian et al. 2023; Pogrzeba et al. 2019). It is also characterised by high biomass, with a dense bundle root system that allows soil penetration over a large area and is capable of growth on many types of soils (Sanderson et al. 2002). The seeds of *D. glomerata* were obtained from Bunnings Pty Ltd in Victoria, Australia.

### Plant uptake

To evaluate the efficacy of the immobilisation treatment on plant uptake of PFAS, each experimental sample was divided into three replicates of around 250 g each. Soil was placed in 360 mL glassware pots with no pores to avoid the possibility of leaching. In addition, three replicates of negative control samples were prepared using a commercial potting mix sourced from Bunnings Pty Ltd in Victoria. In this study, the negative control was used as a baseline for plant development in a standard growth medium. Soil moisture content was adjusted to approximately 20% of the maximum water holding capacity (WHC) for optimum grass growth and maintained throughout the experiment.

The *D. glomerata* grass seeds were sown around 0.5 mm deep in the soil, as instructed by the seed’s supplier. Glass pots were then transferred to a 22 °C growth room with light conditions set at 12 h light/12 h dark cycle throughout the experiment. Pots were distributed on a table and randomised daily to ensure evenness in light and temperature conditions.

The grass was harvested after a growth period of 3 weeks, where the entire plants (roots and shoots) were carefully removed from the soil. The samples were then stored at − 20 °C until further analysis. The harvested plant tissues were first freeze-dried for 24 h at − 85 °C using VirTis Benchtop Pro freeze dryers with Omnitronics.

### Per- and polyfluoroalkyl substances analyses

All PFAS analyses, including total concentration in the soil and plant tissues and leachability, were assessed in the laboratories of ADE Consulting Group Pty Ltd (ADE).

### Per- and polyfluoroalkyl substances analyses from soil

Soil samples were analysed for total PFAS concentration (11 × PFCAs, 5 × PFSAs) as five replicate samples prior to treatment (listed in Table S1). The PFAS was extracted from soil following the protocol described in USEPA 537.1 (EPA 2018) using high-performance liquid chromatography (LCMS/MS, Shimadzu Corp., Kyoto, Japan) coupled to a tandem mass spectrometer triple quadruple operating in negative electrospray ionisation mode and using multiple reaction monitoring (MRM).

### Per- and polyfluoroalkyl substances analyses from the plant

Per- and polyfluoroalkyl substances were extracted from the harvested plant tissues following the same protocol described previously with some modifications (Bräunig et al. 2021b). Grass samples were weighed (dry weight), transferred into a 50 mL polypropylene centrifuge tube, and spiked with 233 µL of surrogate working solution. An aliquot (10 mL) of 1:1 MeOH/ultra-high pure water (methanol HPLC grade) was added, and the mix was shaken by hand for 1 min. An aliquot (20 µL of ammonium hydroxide solution (25%)) was added to each sample to adjust the pH level to 9–10, which is the optimum pH for PFAS extraction from plant tissue. Samples were then shaken by hand for 1 min; this was followed by tumbling for 1 h and centrifugation at 2800 rpm for 10 min. Finally, a syringe and filter (prewashed using MeOH/ultra-high pure water) were used to filter the supernatant into a polypropylene tube. An aliquot of this supernatant (500 µL) was transferred into a sample vial, and 480 µL of 0.58 M acetic acid and internal standard solution (IS) (20 µL) were added. The sample vial was capped and shaken vigorously using a vortex before analysis. For quality assurance and quality control procedures, additional samples were prepared and analysed and can be found in the supplementary material.

All samples were subject to analysis in ADE’s NATA-accredited laboratory for PFAS analysis short-suite to determine PFAS concentrations and bioavailability using liquid chromatography-mass spectrometry (LC–MS).

### Laboratory leaching analysis

The Australian Standard Leaching Procedure (ASLP) was performed to determine the potential desorption of PFAS from the contaminated and treated soil samples under acidic and alkaline conditions. Accordingly, to assess the efficacy of the immobilisation treatment, leachates from the experimental samples were prepared at two pH values, 5 and 7 (Standard 1997). The leached PFAS concentration from the untreated contaminated soil was considered to represent 100% leachability, and the reduction percentage was calculated using the equation below based on this assumption.

$$\mathrm{Percentage reduction}=\left(\mathrm{baseline concentration}-\mathrm{treatment concentration}\right)\times 100$$

where baseline concentration is the PFAS concentration at 0% application rate of the immobilising agent (untreated contaminated soil), which is considered 100% leachability, and treatment concentration is the PFAS concentration at each respective application rate of the immobilising agent.

For the purposes of quality control, a split sample of the collected soil was also delivered to Envirolabs Pty Ltd where a comparative analysis was conducted in conjunction with ADE’s partnered lab, Sydney Laboratory Solutions.

### Ecotoxicity

The ecotoxicity associated with the experimental soil samples was assessed using bioluminescence inhibition testing (the Microtox test) as previously described (Khudur et al. 2015). The acute Microtox reagent containing a freeze-dried marine bacterium *Aliivibrio fischeri* and the reconstitution medium was supplied by JW Industrial Instruments Pty. Ltd. A soil extract was prepared by adding air-dried soil (1 g) of each replicate into 9 mL of water, followed by overnight incubation and centrifugation (twice) at 4500 rpm for 5 min. The luminescence of the test samples was measured using a Microtox® Model 500 Analyser, and the EC_50_ of each replicate was calculated using the provided software (ASTM, 2004). To compare the toxicity level of the experimental soil samples, the toxicity unit (TU) was calculated using the following equation:

$${\text{TU}}=\left(1/{{\text{EC}}}_{50}\right)\times 100$$

### Data and statistical analysis

Bioaccumulation factors (GAF) were calculated for the grass samples analysed. GAFs were calculated using the following equation:

$${\text{GAF}}={C}_{{\text{g}},ww}/{C}_{s,dw}$$

where (*C*_*s*_) the concentrations in soil were calculated for dry soil weight (ng/g dw), while (*C*_*g,ww*_) the concentrations in the grass were calculated as wet weight (ng/g ww) (Bräunig et al. 2019).

Data was subject to analysis of variance (ANOVA) or *T* test using IBM SPSS 28 (IBM SPSS Inc.), as appropriate. In ANOVA, the mean values will be separated using Tukey’s test (*P* = 0.05), where the *F* value will be regarded as significant at *P* > 0.05.

## Results and discussion

### Total per- and polyfluoroalkyl substances

All 5 replicates of representative (prior to treatment) untreated soil samples were assessed for PFAS total concentration (11 × PFCAs and 5 × PFSAs) and PFOS + PFHxS combined concentrations (Table 1). The data of the total concentration of PFAS in untreated soil prior to treatment confirmed that the sum of the mean total concentration of PFAS was 8.05 mg/kg. The PFOS concentration, 7.66 mg/kg, represented around 95% of the total PFAS measured, followed by PFHxS > PFHxA > PFOA > all other PFAS. This level and profile of PFAS contamination have been reported to be typical for sites impacted by 3 M Light Water AFFF (Kabiri et al. 2022; Brusseau et al. 2020; Houtz et al. 2013), with the exception that in these soils, PFHpS was higher than PFOA (Table 1).

**Table 1:** Per- and polyfluoroalkyl substance soil concentrations prior to treatment

PFAS acronym	Full name	Mean total concentration (mg/kg)
PFCA	Perfluoroalkyl carboxylic acids	
PFBA	Perfluorobutanoic acid	0.0062 ± 0.0007
PFPeA	Perfluoropentanoic acid	0.0129 ± 0.0007
PFHxA	Perfluorohexanoic acid	0.08 ± 0.0179
PFHpA	Perfluoroheptanoic acid	0.0036 ± 0.0006
PFOA	Perfluorooctanoic acid	0.0129 ± 0.0027
PFNA	Perfluorononanoic acid	0.0009 ± 0.0001
PFDA	Perfluorodecanoic acid	0.001 ± 0.0001
PFUdA	Perfluoroundecanoic acid	0.0009 ± 0.0001
PFDoA	Perfluorododecanoic acid	0.0011 ± 0.0001
PFTrDA	Perfluorotridecanoic acid	n.d
PFTeDA	Perfluorotetradecanoic acid	n.d
PFSA	Perfluoroalkyl sulfonic acids	
PFBS	Perfluorobutanesulfonic acid	0.0041 ± 0.0002
PFPeS	Perfluoropentanesulfonic acid	0.008 ± 0.0006
PFHxS	Perfluorohexanesulfonic acid	0.2174 ± 0.0155
PFHpS	Perfluoroheptanesulfonic acid	0.0217 ± 0.0022
PFOS	Perfluorooctane sulfonate	7.6627* ± 0.8611
PFAS	Sum of PFAS	8.0481 ± 0.8559
PFHxS + PFOS	Combined	7.8859* ± 0.8639

The results are the means of five replicates (*n* = 5) with the ± standard deviation shown (SD)

*n.d*, not detected

^*^In exceedance of the Defence PFAS Construction and Maintenance Framework Category 2—High Risk minimum soil concentration (LODs and LOQs listed in Table S1)

The PFOS concentration in this study falls within the range reported for firefighting training facility sites reported worldwide (Houtz et al. 2013; McGuire et al. 2014). The combined PFHxS + PFOS mean concentration of 7.89 mg/kg ranged between 95.5 and 99.5% of the total PFAS mass detected in all analysed replicates of the untreated soil and is classified under the Defence PFAS Construction and Maintenance as Category 2—High Risk (1–20 mg/kg). The Defence PFAS Construction and Maintenance Framework classifies PFAS-impacted materials into groups that pose similar risks to the environment and human health (DPFASR 2021). Exceedance of the level of Category 2 does not prevent the reuse of these materials on the site. However, careful consideration and risk assessment would be required to reuse this material.

In addition, when assessed, the PFAS concentration in the contaminated soil prior to treatment exceeds residential land use with garden/accessible soil based on the HEPA PFAS National Environmental Management Plan Version 2.0 (2020), which provides guidance on the management of PFAS-impacted soils (AUSTRALIA and Zealand 2020), as the sum of PFOS and PFHxS concentrations (7.89 mg/kg) exceeds the health investigation level (HIL-A) (0.1 mg/kg). In addition, a mean PFOS concentration of 7.66 mg/kg in the contaminated soil exceeded the ecological indirect exposure value for all land use which is 0.01 mg/kg based on dietary exposure of secondary consumers as the most sensitive exposure pathway assessed (AUSTRALIA and Zealand 2020).

Further, the other PFAS compounds that were dominant in the contaminated soil were the perfluoroalkyl carboxylic acids, PFHxA, ranging from 0.001 to 0.12 mg/kg. Perfluorooctanoic acid (PFOA), the PFCA of concern for NEMP 2.0 (National Environmental Management Plan Version 2.0 (2020)) that provides guidance on the management of PFAS-impacted soils, was detected in all soil replicates, with concentrations ranging between 0.001 and 0.020 mg/kg (Australia And Zealand 2020).

Thus, treating the contaminated soil was required to minimise the risk associated with PFAS contamination. Since leachability is a standard method to assess the potential for PFAS leaching from soil to groundwater, reducing the leachability of PFAS compounds in the groundwater to minimise the movement of PFAS through the environment represents one option.

### Per- and polyfluoroalkyl substances immobilisation and leachability testing

Bench trials were conducted to firstly assess the leachability of PFAS from contaminated soils and secondly to assess the effect of the immobilisation treatment on PFAS leachability at 0.5, 1, 1.5, 2, 3, 4, and 5% RB (w/w) application rate at two pH levels (pH 5 and pH 7).

The total PFAS concentration in the leachate from untreated soil was 145.77 μg/L and 162.90 μg/L at both pH 5 and pH 7, respectively, which was considered 100% leachability from untreated soil (Table 2).

**Table 2:** Data show total per- and polyfluoroalkyl substance concentrations in the leachate at pH 5 and pH 7 trial

Application rate (%) immobilising agent (w/w)	Total leachable PFAS mean concentration (μg/L) at pH 5	Total leachable PFAS mean concentration (μg/L) at pH 7
0	145.77	162.90
0.5	16.74 ± 5.65	143.39 ± 119.6
1	5.92 ± 3.31	11.25 ± 6.5
1.5	1.62 ± 0.49	2.18 ± 0.85
2	1.67 ± 0.24	1.36 ± 0.48
3	0.28 ± 0.12	0.27 ± 0.15
4	0.26 ± 0.09	0.29 ± 0.044
5	0.31 ± 0.15	0.35 ± 0.006

The results are the means of three replicates (*n* = 3) with the ± relative standard deviation shown (R%SD)

**Fig. 1:**
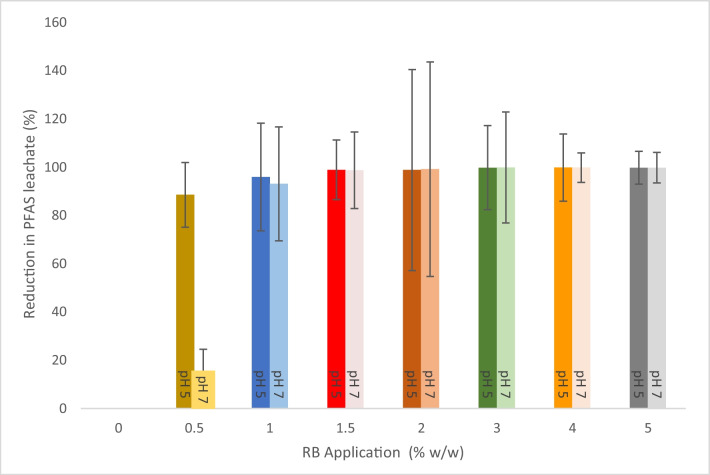
Total PFAS percentage leachate reduction in all experimental samples at pH 5 and pH 7, where the leachate from 0% (w/w) application rate was considered 100% leachability. Data represents as mean (*n* = 3) and error bars represent standard deviation

Overall, the leachability testing of the treated soil samples showed 88.5–99.8% and 11.98–99.84% reduction in the leachability of the total PFAS concentration at pH 5 and pH 7, respectively (Fig. 1).

However, a reduction of 88.5% and 11.98% in the PFAS leachable fraction was recorded at the first dosage rate, 0.5% RB (w/w) at pH 5 and 7, respectively. At 1% RB (w/w) dosage rate, the total PFAS concentration in the leachates was recorded as 5.92 and 11.25 µg/L at pH 5 and 7, respectively (Fig. 1), representing a reduction in leachate compared with the previous application rate. The PFAS leachate fraction reduction was 95.94% and 93.09% at pH 5 and 7, respectively. The PFAS leachate from treated soil samples from 1.5% RB (w/w) application rate showed the maximum reduction, ranging between 99.89% and 98.66% at pH 5 and 7, respectively (Fig. 1).

**Fig. 2:**
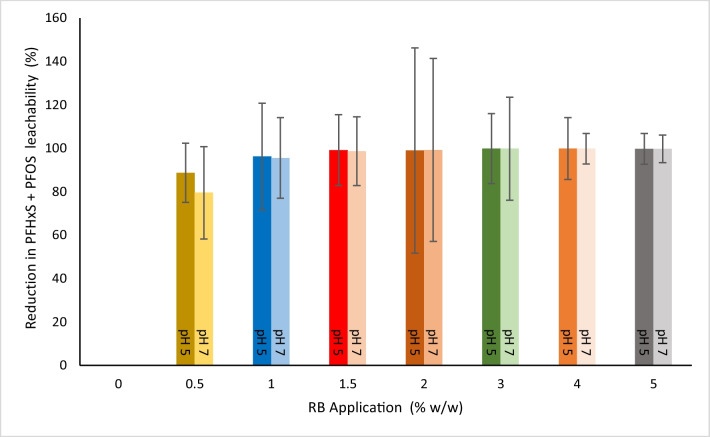
Combined PFHxS and PFOS percentage leachate reduction in all experimental samples at pH 5 and pH 7, where the leachate from 0% (w/w) application rate was considered 100% leachability. Data represents as mean (*n* = 3) and error bars represent standard deviation

For the combined PFHxS + PFOS concentration, the leachability testing of the treated soil samples showed 88.69–99.82% and 76.48–99.83% leachate reduction at pH 5 and pH 7, respectively (Fig. 2).

Similarly, the combined PFHxS and PFOS concentration in the leachate at both pH 5 and pH 7 was 139.13 μg/L and 155.14 μg/L, respectively, considered 100% leachability from the untreated soil. However, a reduction of 88.69% and 76.48% in the combined PFHxS and PFOS leachable fraction was recorded at the first treatment level of 0.5% RB (w/w) at pH 5 and 7. The combined PFHxS and PFOS leachate from treated soil samples from 1.5% RB (w/w) application rate showed maximum reduction ranging between the highest of 99.82% and 99.83% and the lowest of 98.93% and 98.63% at pH 5 and 7, respectively. Both pH 5 and pH 7 trials show a similar pattern in total PFHxS and PFOS percentage leachate reduction in all RB application rates (Fig. 2).

To investigate and assess the effectiveness of the treatment approach, the recommendation provided by the Heads of EPAs Australia and New Zealand (HEPA) PFAS NEPA Plan Version 2.0 (2020) has been applied to PFAS leachate from all experimental samples (Australia And Zealand 2020). The PFAS NEPM 2.0 provides a human health-based guideline for drinking and recreational water.

The immobilisation treatment decreased several values of the combined PFOS + PFHxS mean concentration to below the PFAS NEMP 2.0 recreational water limit (2 µg/L). However, the values of the combined PFOS + PFHxS mean concentration for all experimental samples remained above the PFAS NEMP 2.0 drinking water limit in both pH 5 and pH 7 trials (0.07 µg/L). Starting from the 2% RB (w/w) application rate, the combined PFOS + PFHxS concentration ranged between 1.49 and 0.25 µg/L and 0.26 and 1.23 µg/L at pH 5 and pH 7, respectively, confirming that their concentrations had been reduced to below the PFAS NEMP 2.0 recreational water limit.

Furthermore, NEMP Plan 2.0 (2020) recommends freshwater guideline PFAS values for 99%, 95%, 90%, and 80% species protection (Table 3), which can be defined as levels of environmental protection that should theoretically protect 99, 95, 90, and 80% of species (Australia And Zealand 2020).

**Table 3:** Ecological water quality guideline values recommended in National Environmental Management Plan Version 2.0 (2020)

Exposure scenario	PFOS	Exposure scenario
Freshwater	0.00023 μg/L	99% species protection—high conservation value systems
	0.13 μg/L	95% species protection—slightly to moderately disturbed systems
	2 μg/L	90% species protection—highly disturbed systems
	31 μg/L	80% species protection—highly disturbed systems

The immobilisation treatment applied in this study reduced the combined mean concentration of PFHxS + PFOS at most treatment levels from the 80% ecological protection threshold in the untreated soil in both pH trials (5 and 7). The reduction in the combined PFHxS + PFOS concentration at pH 5 met the NEMP 2.0 90% ecological protection threshold starting from 1.5% RB (w/w) application rates. In contrast, the reduction in the combined PFHxS + PFOS concentration at pH 7 met the NEMP 2.0 90% ecological protection threshold starting from 2% RB (w/w) application rates. The current study showed that the lowest application rate could reduce the combined PFOS + PFHxS concentration (µg/L) below the PFAS NEMP 2.0 Recreational Water limit (2 µg/L) at both tested pH values was 2% RB (w/w) application rate at both pH 5 and 7.

The leachability testing showed significant differences in leachate reduction in all the treated samples compared to the untreated soil at pH 5. The leachability testing conducted at pH 7 demonstrated no significant differences in leachate reduction between the least application rate treatment 0.5% RB (w/w) and the untreated soil. Kabiri et al. in their study showed the effect of pH on the leachability behaviour of PFAS compounds; the leachability of long-chain PFAS was more pH-dependent than short-chain PFAS (Kabiri et al. 2022). Other studies conducted using immobilisation sorbents reported that the reduction in PFAS leachability was affected by many factors, such as initial PFAS concentration and the type of amendment used (Hale et al. 2017; Juhasz et al. 2022b). Another study that used the same absorbent agent as the current study (RemBind®) resulted in an approximately 99.5% decrease in PFAS leachability in comparison to the contaminated untreated soil, highlighting the importance of the uniformity of the stabiliser distribution using a proper soil mixing technique (McDonough et al. 2022).

In addition, Kabiri et al. assessed the effects of factors that might affect the persistence of PFAS binding to the immobilisation absorbent agent (RemBind®), such as extreme temperature, high concentration of competing ions (orthophosphate (H_2_PO_4_^−^) and humic acid (HA)), and high ionic strength. These factors encompass most site conditions; the study therefore confirmed the stability of PFAS binding in soil for extensive periods under conditions expected to change the desorption of PFAS (Kabiri and McLaughlin 2021).

### The effect of adding the immobilising agent on plant uptake of per- and polyfluoroalkyl substances

A plant growth experiment conducted to assess plant uptake of PFAS from the experimental soil samples showed a high concentration of PFAS taken up by the plant from untreated soil while a significant reduction in plant uptake was observed from treated soil. Out of 16 PFAS compounds investigated in this study, 11 were detected in the grass from the untreated soil, whereas only 7 PFAS compounds were detected from the grass in the treated (0.5–5% RB w/w) soil. The PFAS compounds, PFBA, PFUdA, PFDoA, PFTrDA, and PFTeDA were not detected in plant tissues in all experimental samples. The average PFAS concentration in plant tissue from all experimental samples is listed in Table S2.

The sum of PFAS concentration in the plant tissues showed the highest concentration of 5632 μg/kg in the untreated soil. All the treated soils showed a significant decrease in the plant uptake of PFAS, with PFAS concentrations in the plant tissues ranging from 451.7 to 1122.9 μg/kg in all treated soil samples. The combined PFHxS and PFOS in the plant tissues harvested from the untreated soil showed the highest concentration of 5322.3 μg/kg, while the plant tissue concentrations of the combined PFHxS and PFOS were reduced by 5–12-fold in all treated soils. The combined PFHxS and PFOS ranged between 94.5 and 98.4% of the total PFAS mass detected in all plant tissue samples (Table S2).

**Fig. 3:**
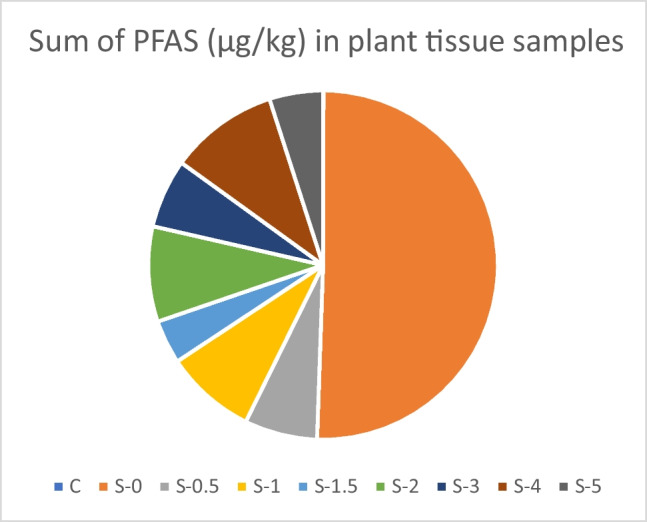
PFAS soil concentrations in freeze-dried plant tissue samples (µg/kg). C, control; S-0, untreated contaminated soil; S-(0.5, 1, 1.5, 2, 3, 4, 5), (0.5, 1, 1.5, 2, 3, 4, 5) % (w/w) Remind® application rate respectively (data is presented as mean, *n* = 3)

Among the 16 PFAS investigated in this study, PFOS was the dominant PFAS compound detected in all plant tissue, with the highest of 4820.2 μg/kg from untreated soil and between a 5- and tenfold reduction in the uptake from treated soil (Fig. 3). The second dominant PFAS concentration detected in the harvested plant tissues was for the perfluoroalkyl sulfonic acid (PFSA), PFHxS with the highest of 502.1 μg/kg from untreated soil and the lowest of 36.93 μg/kg from 2% RB (w/w) treated soil (Fig. 3).

Overall, a reduction of between 5- and 11-fold in grass PFAS uptake at all application rates was observed. Out of 14 PFAS compounds detected in the soil, 11 were detected and showed the same profile in the harvested grass from untreated soil. Perfluorooctane sulfonate contributed 95% of the total PFAS, followed by PFHxS > PFHxA > PFHpS > PFHpS and all other PFAS. The grass did show impaired growth in treated soil at all application rates compared to the untreated soil, showing less biomass and bale grass colour. This could be due to the binding of the amendment to essential nutrients necessary for plant growth as previously described (Bräunig et al. 2021a; Bonaglia et al. 2019). In practice, this can be rectified by adding nutrient fertilisers with sorbent if required.

Most studies have focused on how PFAS affects human health through consuming contaminated food. Per- and polyfluoroalkyl substances in the soil not only pose a direct contact risk to humans but also to the ecosystem through leaching. In this study, the focus was on how much PFAS in contaminated soil can be taken up by grass, which can be ingested by animals at a higher trophic level and may magnify through the food chain to a level that poses a risk to humans. The combined PFHxS + PFOS concentration in the grass from untreated soil exceeded the NEMP 2.0 biota guideline value for direct ecological exposure for mammalian diet consumption of biota as wet-weight food (4.6 μg/kg)(Australia and Zealand 2020). However, the combined PFHxS + PFOS concentration of grass in treated soil at all application rates was reduced below the NEMP 2.0 mammalian diet consumption of biota threshold (4.6 μg/kg). In comparison, in PFAS plant uptake, 11 PFAS were detected in the grass from the untreated 0% RB (w/w) soil out of the 14 detected in the soil. Perfluorooctane sulfonate contributed 95% of the total PFAS, followed by PFHxS > PFHxA > PFHpS > PFHpS and all other PFAS. PFAS was not detected in the grass from the control soil samples.

**Fig. 4:**
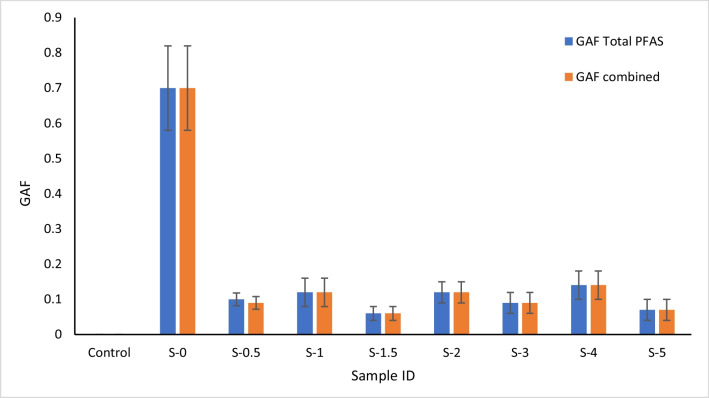
The accumulation factors (GAF) of total PFAS and combined PFOS + PFHxS in the experimental samples (data is presented as mean, *n* = 3, and error bars represent standard deviation)

To compare the impact of the immobilisation treatment on the PFAS concentration in plant tissues, the accumulation factors (GAF) were calculated (Fig. 4). GAF is the ratio between the concentration of PFAS taken up by the plant to the concentration of PFAS in the soil.

Accumulation factor (GAF) values were significantly decreased in all RB application rates up to tenfold, although there were no significant differences between the treatments of different application rates.

Other studies have shown that the GAF values were higher as the PFAS concentration in soil increased. Similarly, the addition of immobilising sorbents caused a reduction in the accumulation factor (GAF) in wheatgrass (Bräunig et al. 2021a; Juhasz et al. 2022b).

### Ecotoxicological assessment

**Fig. 5:**
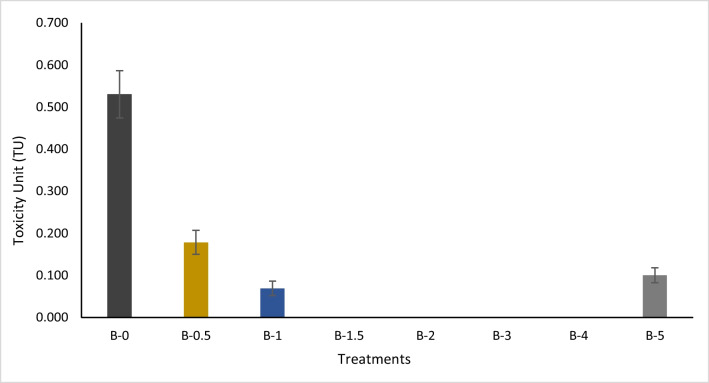
Toxicity unit (TU) values associated with the experimental soil samples at 10 min using the Microtox test (data is presented as mean, *n* = 3, and error bars represent standard deviation)

The Microtox test was conducted in this study to assess the ecotoxicity associated with the untreated and treated (0.5, 1, 1.5, 2, 3, 4, and 5% RB w/w) soil samples. The (EC_50_) values recorded at 10 min were transformed into toxicity unit (TU) to compare the experimental sample’s toxicity (Fig. 5). Based on this scale, the toxicity of soil samples can be classified as no toxicity (TU = 0), moderate toxicity (TU < 1), or high toxicity (TU = 1–10) (Khudur et al., 2018).

Although the data showed relatively low toxicity in all samples, the untreated soil showed significantly higher toxicity compared to the treated soil. The toxicity of PFAS was reduced at least threefold by the RB treatment. A significant reduction in toxicity was recorded in all RB application rates where the TU values were 0.178 at 0.5% RB (w/w), 0.100 at 5% RB (w/w), and 0.069 TU at 1% RB (w/w). There was no toxicity recorded at 1.5–4% RB (w/w) treatments. Further, no significant differences were recorded in the ecotoxicity of application rates 1–5% RB (w/w). Earlier studies conducted on the toxicity of PFAS using earthworms showed a decrease in the average weight of the earthworm incubated on immobilised treated soil samples during the experiment in comparison to the untreated PFAS-contaminated soil (Bräunig et al. 2021a). To the best of the authors’ knowledge, this is the first study on the toxicity of PFAS-contaminated soil using the Microtox test. Another study investigated the bioaccumulation of PFAS in earthworms living on contaminated soil for the same experimental period (28 days) with no recorded weight loss (Rich et al. 2015). The authors concluded that the soil amendment may bind to the nutrients in this soil, inhibiting assimilation and growth rather than a result in increasing toxicity of PFAS-contaminated soil after treatment (Bräunig et al. 2021a). In the current study, poor plant growth was observed in the contaminated and treated sample in comparison to the control. However, this can be easily rectified in practice by the addition of nutrient fertilisers.

Also, for comparison, the toxicity of RB was also tested using Microtox. Although the application rates recommended by the company were ranged between 0.5 and 5%, the toxicity of up to 20% of RB was tested in this study. The Microtox test shows no toxicity associated with RB (2.5, 5, 10, and 20%). In addition, for the same purposes, the toxicity of 20, 10, 5, 2.5, and 1% of PFAS solution suspended in water was also tested using the Microtox test. The solution consisted of PFBA, PFBS, PFHxA, PFHxS, PFOA, and PFOS with concentrations of 15.6, 25.6, 23.6, 32.4, 23, and 10.8 mg/L, respectively, in the 20% samples. The 10 min toxicity unit (TU) of 20% and 10% was 3.12 and 0.46, respectively, while no toxicity was recorded at 1, 2.5, and 5%.

## Conclusion

This study has shown the effectiveness of an immobilisation approach using an immobilising agent at different application rates (0.5, 1.0, 1.5, 2.0, 3.0, 4.0, and 5.0% w/w) in treating PAFS-contaminated soil. The addition of the immobilising agent to the PFAS-contaminated soil significantly reduced PFAS leachability, bioavailability, and associated ecotoxicity. The leachability of PFAS was reduced by 88.5–99.8% in terms of total PFAS leachability and 88.7–99.8% in terms of combined PFOS and PFHxS leachability in the treated soil samples compared to untreated soil samples at pH 5. Similarly, a reduction in the range of 12.0–99.8% in total PFAS leachability and 79.5–99.9% in combined PFOS and PFHxS leachability was observed at pH 7. Plant uptake was also significantly decreased in both total PFAS and combined PFOS and PFHxS concentrations by around 5–12-fold in all treated soil samples compared to the untreated soil. In addition, the application of the immobilisation agent reduced the associated ecotoxicity by at least threefold. The results from this laboratory study show that among the application rates applied in this study, the 2% RB (w/w) application rate would be recommended as an optimum treatment option for immobilising PFAS in contaminated in the current soil as it resulted in approx. 99% reduction in PFAS; further field-based studies should be carried out. In addition, temporal studies looking at the effect of time on the capacity of the immobilising agent to limit PFAS leaching out are required to evaluate the immobilised PFAS in terms of potential risks to the ecological system in the future.

### Supplementary Information

Below is the link to the electronic supplementary material.

Supplementary file1 (DOCX 28 KB)
