# Supplementary material for: Assessing the impact of immobilisation on the bioavailability of PFAS to plants in contaminated Australian soils
Source: Environ Sci Pollut Res Int. 2024 Feb 19;31(13):20330–42. doi: 10.1007/s11356-024-32496-7 (PMC10927784; doi:10.1007/s11356-024-32496-7)
Supplement: Supplementary file 1 — Supplementary file1 (DOCX 28 KB) [file 11356_2024_32496_MOESM1_ESM.docx]

**Table S1:** Full compound list and CAS numbers for investigated PFAS, PFAS total concentration and Australian Standard Leaching Procedure Total Concentration (TC) and Total Concentration Leachate Procedure (TCLP) limit of reporting.

| **Per-fluoroalkyl carboxylic acids and sulfonic acids** | | | **Laboratory Analysis limit of reporting** | | | | |
| --- | --- | --- | --- | --- | --- | --- | --- |
| **Acronym** | **Full name** |  | **SLS** | **SLS** | **Envirolab** | **Envirolab** |  |
| **PFCAs** | **Per-fluoroalkyl carboxylic acids** | **CAS Number** | **TC (mg/kg)** | **TCLP** | **TC (mg/kg)** | **TCLP** |  |
| **PFBA** | Perfluorobutanoic acid | 375-22-4 | 0.004 | 0.1 | 0.0002 | 0.02 |  |
| **PFPeA** | Perfluoropentanoic acid | 2706-90-3 | 0.004 | 0.1 | 0.0002 | 0.02 |  |
| **PFHxA** | Perfluorohexanoic acid | 307-24-4 | 0.001 | 0.01 | 0.0001 | 0.01 |  |
| **PFHpA** | Perfluoroheptanoic acid | 375-85-9 | 0.001 | 0.01 | 0.0001 | 0.01 |  |
| **PFOA** | Perfluorooctanoic acid | 335-67-1 | 0.001 | 0.01 | 0.0001 | 0.01 |  |
| **PFNA** | Perfluorononanoic acid | 375-95-1 | 0.001 | 0.01 | 0.0001 | 0.01 |  |
| **PFDA** | Perfluorodecanoic acid | 335-76-2 | 0.001 | 0.01 | 0.0005 | 0.02 |  |
| **PFUdA** | Perfluoroundecanoic acid | 2058-94-8 | 0.001 | 0.01 | 0.0005 | 0.02 |  |
| **PFDoA** | Perfluorododecanoic acid | 307-55-1 | 0.001 | 0.01 | 0.0005 | 0.05 |  |
| **PFTrDA** | Perfluorotridecanoic acid | 72629-94-8 | 0.001 | 0.01 | 0.0005 | 0.1 |  |
| **PFTeDA** | Perfluorotetradecanoic acid | 376-06-7 | 0.001 | 0.01 | 0.005 | 0.5 |  |
| **PFSAs** | **Per-fluoroalkyl sulfonic acids** |  |  |  |  |  |  |
| **PFBS** | Perfluorobutanesulfonic acid | 375-73-5 | 0.001 | 0.1 | 0.0001 | 0.01 |  |
| **PFPeS** | Perfluoropentanesulfonic acid | 2706-91-4 | 0.001 | 0.01 | 0.0001 | 0.01 |  |
| **PFHxS** | Perfluorohexanesulfonic acid | 355-46-4 | 0.001 | 0.01 | 0.0001 | 0.01 |  |
| **PFHpS** | Perfluoroheptanesulfonic acid | 375-92-8 | 0.001 | 0.01 | 0.0001 | 0.01 |  |
| **PFOS** | Perfluorooctanesulfonic acid | 1763-23-1 | 0.001 | 0.01 | 0.0001 | 0.01 |  |
| **PFAS Sums** |  | | | | | | |
| **Sum of PFAS** |  | | N/A | N/A | 0.00001 | 0.01 |  |
| **Sum of PFHxS and**  **PFOS** |  | | N/A | N/A | 0.00001 | 0.01 |  |

| **Table S2**: PFAS soil concentrations in freeze-dried plant tissue samples (µg/kg) C= Control, S-0 = untreated contaminated soil, S-(0.5, 1, 1.5, 2, 3, 4,5) = (0.5, 1, 1.5, 2, 3, 4,5) % (w/w) Remind application rate respectively (Data is presented as mean, *n*=3) | | | | | | | | | |
| --- | --- | --- | --- | --- | --- | --- | --- | --- | --- |
| PFAS  Acronym | Total per- and polyfluoroalkyl substances mean concentration (μg/kg) in plant tissue samples | | | | | | | | |
|  | **C** | **S-0** | **S-0.5** | **S-1** | **S-1.5** | **S-2** | **S-3** | **S-4** | **S-5** |
| PFBA | n.d | n.d | n.d | n.d | n.d | n.d | n.d | n.d | n.d |
| PFPeA | n.d | 48.5 | 3 | 2.2 | 1.4 | 2.3 | 2.2 | 3.8 | 2.3 |
| PFBS | n.d | 14.7 | n.d | n.d | n.d | n.d | n.d | n.d | n.d |
| PFHxA | n.d | 169.9 | 4.7 | 9.5 | 3 | 9.4 | 7.2 | 8.5 | 4.5 |
| PFPeS | n.d | 21.5 | n.d | n.d | n.d | n.d | n.d | n.d | n.d |
| PFHpA | n.d | 4.5 | n.d | n.d | n.d | n.d | n.d | n.d | n.d |
| PFHxS | n.d | 502.1 | 25.1 | 35.7 | 17.7 | 36.9 | 29.4 | 36.8 | 20.5 |
| PFHpS | n.d | 30.2 | 2.9 | 3.2 | 2.5 | 3.6 | 1.2 | 4.2 | n.d |
| PFOA | n.d | 18.8 | 1.4 | 2.2 | 1.1 | 2 | 3 | 2.1 | 2.1 |
| PFOS | n.d | 4820.2 | 715.1 | 884.6 | 425.9 | 929.4 | 669.1 | 1067.5 | 523.8 |
| PFNA | n.d | 1.3 | n.d | n.d | n.d | 0.3 | n.d | n.d | n.d |
| PFDA | n.d | 0.3 | n.d | n.d | n.d | n.d | n.d | n.d | n.d |
| PFUdA | n.d | n.d | n.d | n.d | n.d | n.d | n.d | n.d | n.d |
| PFDoA | n.d | n.d | n.d | n.d | n.d | n.d | n.d | n.d | n.d |
| PFTrDA | n.d | n.d | n.d | n.d | n.d | n.d | n.d | n.d | n.d |
| PFTeDA | n.d | n.d | n.d | n.d | n.d | n.d | n.d | n.d | n.d |
| Sum of PFAS in µg/kg | 0 | 5632.1 | 752.2 | 937.4 | 451.7 | 983.9 | 712.2 | 1122.9 | 553.3 |
| PFHxS + PFOS in µg/kg | 0 | 5322.36 | 740.15 | 920.34 | 443.62 | 966.31 | 698.54 | 1104.30 | 544.32 |

For quality assurance and quality control procedures, additional samples were prepared and analysed:

- Two blank samples: one analyte free spiked with Internal Standard and Surrogate solution.
- Laboratory Control Sample (BSP): analyte free spiked with Internal Standard, Surrogate and 1.50 $\mu$L of Analytical Spiking Solutions.
- Matrix Spike (MSP): in which a sample was randomly selected from the batch and fortified with Analytical Spike Solution.
- Two duplicate laboratory samples: A sample was randomly chosen and prepared in duplicate.
